# The New First-in-Human EMA Guideline: Disruptive or Constructive? Outcomes From the First EUFEMED Discussion Forum

**DOI:** 10.3389/fphar.2019.00398

**Published:** 2019-04-24

**Authors:** Kerstin Breithaupt-Grögler, Tim Hardman, Jan de Hoon, Yves Donazzolo, Sylvie Rottey, Hildegard Sourgens, Steffan Stringer

**Affiliations:** ^1^Association for Applied Human Pharmacology, Hamburg, Germany; ^2^Association for Human Pharmacology in the Pharmaceutical Industry, London, United Kingdom; ^3^Niche Science & Technology Ltd., Richmond, United Kingdom; ^4^Belgian Association of Phase-1 Units, Brussels, Belgium; ^5^Association Francaise de Pharmacologie Translationnelle (Formally Le Club Phase 1), Lyon, France

**Keywords:** early phase clinical drug development, revised first-in-human guideline, conference report, EMA (European Medicines Agency), guidelines, EUFEMED, discussion

## Abstract

The European Federation for Exploratory Medicines Development (EUFEMED) organized a meeting in Leuven, Belgium entitled ‘The new FIH EMA guideline: Disruptive or constructive?’ to provide a forum for stakeholders to discuss the guideline’s operational impact. The revised EMA Guideline on strategies to identify and mitigate risks for first-in-human (FIH) and early clinical trials with investigational products was published on 20 July 2017. The revision gave guidance on sentinel dosing/staggering of subjects within a multiple-ascending dose (MAD) clinical trial, permissible maximum exposure/investigation of supra-therapeutic doses and dose escalations above the no-observed adverse effect level. As the guidelines came into operation on 1 February, 2018 it was assumed that by the date of the meeting many early phase stakeholders had gathered sufficient first-hand experience of working within the guideline to discuss their thoughts on its impact. The concluding part of the meeting focused on the possible differences between European countries in handling the revised FIH guideline and ways of achieving harmonization. Information on current industry practice was gathered by online polling during the meeting, where perception of the revised guideline as either ‘disruptive’ or ‘constructive’ was explored at the start and at the end of the Forum along with recommendations on reducing future regulatory discordance. It was generally agreed that the necessary changes encompassed by new guidelines included both constructive and disruptive aspects. The final vote on whether the new FIH guideline is disruptive or constructive was taken by 69 delegates: 51% stated that it was both constructive and disruptive, 48% decided on constructive, none on disruptive and 1% were still undecided. It was generally accepted that stakeholders need to continue in a process of stakeholder engagement and discussion, particularly on critical safety issues. Such an approach allows partners to adopt a proactive approach to sharing best practice. For example, attendees agreed that a ‘Question and Answer’ document harmonized between the European agencies is required for the sentinel approach and for the selection of supratherapeutic doses.

## Introduction

The European Federation for Exploratory Medicines Development (EUFEMED^1^) is a not-for-profit association that aims to improve the early phase clinical drug development process in Europe (Van Bortel et al., 2018). On the 19 September 2018, EUFEMED organized a meeting in Leuven, Belgium entitled ‘The new FIH EMA guideline: Disruptive or constructive?’ to provide a forum for stakeholders to discuss the guideline’s operational impact. The revised EMA Guideline on strategies to identify and mitigate risks for First-in-Human (FIH) and early clinical trials with investigational products was published on 20 July 2017 (European Medicines Agency Science Medicines Health, 2017). The revision gave guidance on sentinel dosing (where one person in a first cohort of participants receives a single dose of investigational product in advance of the full study cohort) and the staggering of subjects (that includes a specified follow-up interval between administration of the product to a subject, or small group of subjects, and administration to the next subject or group of subjects) within a multiple-ascending dose (MAD) clinical trial. The guideline also considers permissible maximum exposures and the use of supra-therapeutic doses as well as dose escalations that extend beyond the predicted no-observed adverse effect level. As the guidelines came into operation on 1 February, 2018 it was assumed that by the date of the meeting many early phase stakeholders had gathered sufficient first-hand experience of working within the guideline to discuss their thoughts on its impact.

The event welcomed over 100 attendees. The aim was for the audience to drive the program through the use of active directed polling employing the *sli.do* online tool^2^. The audience were initially asked to provide feedback on their areas of specific expertise: 51% worked in contract research organizations or as consultants; 38% were sponsors (from pharmaceutical companies) or investigators; 11% represented competent authorities or ethics committees. Participants were also asked at the outset whether they felt that the revised FIH guideline was disruptive or constructive: 35% of the responders indicated that the guidelines were constructive, 1% disruptive, 47% both constructive *and* disruptive and 16% felt that they didn’t know enough to comment. Electronic balloting of attendees continued throughout the meeting, with the organizers seeking their opinion, understanding and insights regarding the guidelines as well as any difficulties they may have experienced when they first started working with the updated recommendations.

Prior to the meeting, the membership of EUFEMED and other stakeholders involved in early medicines development were canvassed for their opinion on various aspects of the new EMA guideline. The assessment was conducted in the form of two online polls, one was conducted by the French Club Phase I association in September 2017 (Sourgens and Donazzolo, 2018b) and the other by EUFEMED in March 2018. The polls were similar but not identical in format (Sourgens and Donazzolo, 2018a).

French regulatory authorities introduced new regulatory guidelines in March 2016 following the BIA 10-2474 incident that occurred in January 2016, where a participant in a Phase I trial died. Provisions made in terms of stopping rules were included in the revised EMA FIH guideline. The impact of the change in regulations was addressed in an online survey of early development stakeholders across France in September 2017 (Sourgens and Donazzolo, 2018b). Of those responding, 39 considered themselves to be active and experienced in early medicines development: of these, fewer than 5% felt that the revised guideline could be considered ‘very clear’; approximately 90% felt little need to make anything more than minor changes to methods used for the identification of starting doses; more than 80% reported no or minor changes in the definition of stopping rules and the sentinel approach. Areas of most concern included:

1. Difference of interpretation of the revised guideline between countries;
2. Identification/selection of animal species relevant for pharmacology AND toxicity AND metabolism;
3. Clarification of investigations needed regarding metabolites in toxicology and safety pharmacology studies;
4. Top dose: evaluation of supra-therapeutic doses;
5. Defining meaningful target pharmacodynamic changes in healthy subjects;
6. Limits to maximum exposure;
7. Clear requirements for determining maximum dose: exclusivity of pharmacodynamic and/or pharmacokinetic data; and
8. Increasing doses in early phase trials beyond the animal no-observed-adverse-effect level (NOAEL).

The impact of the implementation of the revised guideline on early clinical development in the European Union was rated as neutral to positive by more than 80% of the French responders.

The survey of the EUFEMED membership was submitted to approximately 1,000 candidates, 125 of who provided a response (Sourgens and Donazzolo, 2018a). Overall, only minor differences emerged between the findings of the French and the European surveys. Most responders were based in Western parts of Europe and represented sponsors and investigators or were employed by contract research organizations. The majority (88%) indicated that they were well experienced in FIH trials: 72% reporting having conducted more than 10 FIH trials over the last 10 years, including clinical trials with first-in-class compounds, small molecules, biologics and well-known medicines. It was therefore concluded that any findings from the survey would represent the opinions and experiences of highly motivated and educated stakeholders in early clinical development. Of the survey responders, 31% felt that the revised guideline was ‘very clear’; 74% acknowledged little more than minor changes in the selection of the starting dose and 68% reported little or no change to the definition of stopping rules (for individuals and cohorts). However, more than 90% felt that the totality of revised instructions represented a marked change to current practice. Aspects of the guidelines that elicited most interest involved sentinel dosing, definition of maximum exposure, decisions on dose escalation steps and understanding of non-clinical requirements. Overall, 80% of responders indicated that the impact of the implementation of the revised guideline on early clinical development in the EU was neutral to positive.

## Viewpoints and Experience of European Regulatory Agencies

Representatives from six European regulatory agencies and the European Medicines Agency (EMA) provided the audience with their perspectives of the implementation of the revised guideline. Their briefing in preparation for the meeting comprised the following requests for elucidation on their current position:

1. Percentage of FIH clinical trials requiring refusal because of non-adherence to the revised guideline, ethical constraints;
2. Percentage of FIH trials NOT requiring any comments, approvable and full in line with the revised FIH guidance;
3. Do you experience other concerns not addressed in the survey?

### European Medicines Agency

*Stefano Ponzano* from the Clinical Pharmacology and Non-clinical Support Office of the EMA commented on recent experience of questions submitted to the agency for scientific advice on FIH clinical trials (Stefano, 2018). He noted that the number of requests for advice from the agency has been decreasing since the publishing of the guideline, though this observation was based on the limited data. Reviewing the last 16 requests submitted to the EMA since 2017, where questions were put to the Committee for Medicinal Products for Human Use (CHMP) about FIH trials, the most frequently submitted questions related to the design of FIH clinical trials (12 of 27 questions). The main topics covered target populations, endpoint selection, dose level identification, study stopping criteria and safety monitoring as well as which criteria are required to move from a FIH clinical trial in healthy volunteers to a first in patients trial under integrated protocols.

Some examples of recommendations provided by the CHMP through the scientific advice procedures, included the necessity for sponsors to provide additional data from *in vitro* non-clinical studies to better define proof of concept and to underline the relevance of the animal species used in the non-clinical studies with regards to preparing for clinical trials. Based on the level of uncertainty, sponsors were advised to consider not only the NOAEL but also the minimal anticipated biological effect level [MABEL] and the pharmacologically active dose [PAD] when calculating starting doses. The CHMP recommend providing justification when sponsors want to investigate doses beyond the predicted pharmacodynamic range. In particular, the agency request a clear rationale for proposed doses beyond the envisaged maximum dose as well as proposing less aggressive dose escalation strategies depending on the level of uncertainty of the investigational medicinal product.

In concluding his presentation, Stefano Ponzano highlighted that his assessment was based on a small sample making it difficult to assess the extent to which the revised guideline has impacted on the type or number of requests being submitted to the agency. He did, however, express the importance of achieving harmonization of FIH decisions provided by the various European agencies. Dr. Ponzano recalled several initiatives that the EMA are currently involved with to achieve closer working standards, such as a non-clinical training curriculum that is currently being developed by the EU Network Training Centre. The curriculum will include webinars for non-clinical assessors related to the FIH guideline. The Clinical Trial Facilitation Group (CTFG) in collaboration with the EU NTC organized a workshop in March 2017 for non-clinical and clinical assessors on the revision of the FIH guideline. The Clinical Trial Regulation, which entered into force on 16 June 2014, identifies the EMA as being responsible for developing and maintaining the clinical trial portal and database in collaboration with the Member States and the European Commission. Stakeholders will have access to the submission of clinical trial applications and authorizations within the EU once the single EU portal becomes active. It is envisaged that this will be an important tool in identifying possible issues linked to the implementation of the new guideline. A close interaction between the EMA and the various competent authorities remains key to achieving harmonization. A general but important remark made was that the guideline is driven by science, it is a tool to help develop a safe and correct clinical trial. It remains imperative to provide thorough scientific information in the reports and submission documents, ensuring the safety of the subjects.

### Medicines and Healthcare Products Regulatory Agency, United Kingdom

*Kirsty Wydenbach*, Senior Medical Assessor and Deputy Unit Manager CTU at the UK Medicines & Healthcare products Regulatory Agency (MHRA) reported on the numbers of FIH clinical trials conducted in the United Kingdom (Wydenbach, 2019). Records indicate that 20 – 25% of all FIH clinical trials in the EU have some UK involvement. Reviewing the available data, it seems that there has been no change in the number of ‘Grounds for Non-Acceptance’s’ (GNAs) issued by the agency since the implementation of the revised guidance and no rejections due to non-compliance with the revised guideline. Overall, the rate of Phase I rejections issued by the MHRA is in line with all other phase trials.

Surprisingly, most rejections resulted from sponsors not responding at all to GNAs (five of eight). Reasons for the provision of the GNAs included: absence of communication plans for multi-site trials; failure to provide a maximum dose or maximum number of cohorts; unclear individual stopping rules or rules for stopping specific parts of the trials or the whole trial; poor consideration of the potential for drug interactions in protocols that include combination therapy or concomitant medications (ignoring Summary of Product Characteristics risks for marketed products); contraception not aligned with CTFG guidance and inadequate pregnancy testing; proposed healthy volunteers doses far above predicted therapeutic range without any clear justification being provided; no clear characterization of analytical assays used in non-clinical pharmacokinetic and toxicokinetics; lack of quality data. No trend in the issuing of GNAs was apparent when assessed by product type (small molecular entities or biologics).

None of the examples provided were considered by the MHRA to be particularly specific to FIH trials. These findings reflected those reported in the ‘Common issues identified during clinical trial applications’ recently published on the MHRA website (Medicines and Healthcare Products Regulatory Agency, 2018). In terms of issues such as dose ranges in healthy volunteers that extend beyond the therapeutic range, Dr. Wydenbach noted that the MHRA requires sponsors to provide justification for their dosing strategy and provide sufficient data to thoroughly assess their position. She also noted that protocols that include adaptive elements are welcomed by the MHRA, provided they are clearly defined upfront. Inclusion of adaptive elements at a later date may be accepted as long as any supporting rationale is based on emerging data. Sentinel dosing is not seen universally across trials and it is not a required aspect for trials in United Kingdom; the MHRA feel that it is acceptable not to apply a sentinel dosing strategy when a suitable justification is provided.

In concluding, Dr. Wydenbach stated that the implementation of the revised FIH guideline has had no noticeable impact on submissions to or approvals issued by the MHRA. Expectations remain unchanged for the future. According to her experience, the biggest barrier to innovation is not seeking advice from a regulator early enough or at all.

### Bundesinstitut für Arzneimittel und Medizinprodukte, Germany

*Sarah Heil*, Clinical Assessor at Bundesinstitut für Arzneimittel und Medizinprodukte (BfArM, Germany), emphasized that the revised FIH guideline underlines how safety should not be compromised in the interests of speed of acquiring data or for logistical reasons. Thus, risk mitigation activities should be proportionate to the degree of uncertainty and the potential risks identified.

#### Maximum Exposure

Sarah Heil noted that maximum exposure must take non-clinical data into account and must not exceed the NOAEL recorded in the most sensitive species. Healthy subjects should not be exposed to doses exceeding the anticipated therapeutic dose without a clear rationale (ATD). The maximum tolerated dose (MTD) needs to be well defined in patients, whereas it is regarded as unethical to assess the MTD in healthy subjects.

#### Pharmacokinetic-Pharmacodynamic Data for Dose Escalation

The information required in the protocol and the Investigator’s Brochure should include sufficient data on:

(i) Primary and secondary pharmacodynamics;
(ii) Mode of action;
(iii) Interaction with ‘off-targets’ (e.g., secondary pharmacodynamics);
(iv) Pharmacokinetics and toxicokinetics of all available species; and
(v) Specification of dose steps depending on non-clinical data (toxicity or effect).

Grounds for non-acceptance: Reasons for the provision of GNAs issued by the BfArM are categorized in Table 1. In general, BfArM sees key aspects of the study design as including choice of study population, giving due consideration of aspects such as subject age, and the description of what defines a healthy subject. They rarely issue objections relating to number of subjects for study, interval between dosing and dose transition. Most common requested changes include clarification of stopping criteria, missing sentinel approach, dosing justification and inclusion criteria (Breithaupt-Groegler et al., 2017).

**Table 1:** Categorical reasons for the provision of GNAs issued by the BfArM.

Very rare objections	Regular objections	Critical assessments
First/starting dose Dose escalation increments	Inclusion criteria (who is a healthy subject)	Transition to next dose increment/cohort/next study part requires a substantial amendment; pharmacokinetic/pharmacodynamic to be analyzed before proceeding
Maximum dose/exposure	Dosing justification insufficient	Pharmacokinetic/pharmacodynamic half-life of the investigational medicinal products and therefore washout times if the same subjects are participating in multiple cohorts/accumulation for multiple dosing parts
Maximal duration of treatment	Missing sentinel approach	Also for early phase trials with ‘well-known substances’ consideration of the ‘safety factor’
Route and rate/frequency of administrations	Safety (and/or effect) parameters to monitor and intensity of monitoring (e.g., ECG, further laboratory parameter, pregnancy test)	
Number of subjects included in study cohorts	Implementation of stopping criteria inappropriate	


*ECG, Electrocardiogram*.

### Centrale Commissie Mensgebonden Onderzoek, Netherlands

Table 2 provides a summary of the essential changes in the revised EMA FIH guideline previously noted by *Joop van Gerven*, Chairman of the Central Committee on Research Involving Human Subjects (CCMO, Netherlands) (Bonelli and Van Gerven, 2018) and discussed during the meeting.

**Table 2:** A summary of the essential changes in the revised EMA FIH guideline.

More emphasis on pharmacology and action mechanism	More emphasis on protocol arrangements	More emphasis on ‘totality of evidence’
MABEL, pharmacokinetic, pharmacodynamic, PAD, ATD (no MTD)	Rationale / justification Predefined expectations	Structured IB assessment (ib-derisk.org) (European Medicines Agency Pre-authorisation Evaluation of Medicines for Human Use, 2005)
Primary / secondary pharmacology	Amendments	Translational models, Pharmacokinetic/pharmacodynamic
Pharmacokinetic and pharmacodynamic monitoring	Stopping rules Responsibilities	Decisions based on integration of emerging data
Dosing limits


*MABEL, minimal anticipated biological effect level; PAD, pharmacologically active dose; ATD, anticipated therapeutic dose; MTD, maximal tolerated dose.*

*Joop van Gerven* highlighted how it is important to provide a thorough description of the pharmacology and the mechanism of action (MoA) of investigational medicinal products (IMPs) within the protocol. He noted that insufficient emphasis is currently being placed on collecting data that can define the MoA of new agents. Sponsors also seem to put less effort into researching, understanding and reporting the secondary pharmacology characteristics. Equally, issues surrounding pharmacokinetic and pharmacodynamic monitoring appear to be limiting the amount of information emerging from non-clinical pharmacology studies. And yet, most clinical pharmacologists working for regulatory agencies are interested in receiving as much of this type of information as possible. Not only does such data help Sponsors make predictions of possible ‘off target’ effects and their consequences, it also helps investigators and regulators understand what is happening or happened if the drug doesn’t work or if things go wrong (Van Gerven and Cohen, 2018).

The CCMO has seen a philosophical change in its approach to FIH clinical trials. Beyond the emphasis on pharmacology and MoA, it is placing greater emphasis on the protocol itself, requiring sponsors to provide justification for various strategies, providing predefined expectations and stopping rules as well as defining responsibilities. Effectively, this translates into the adoption of a ‘totality of evidence’ approach to achieving study approval that is based on integration of emerging data. Much of the decision process after submission of a protocol is devoted to clarification of these issues during questions and answers.

Summarizing the experience of FIH submissions in Netherlands, Joop van Gerven noted that, thus far, very few studies have been rejected by CCMO since the introduction of the revised guideline in 2017. However, clarifications and/or modifications related to the above were often required before approval. Where rejections occurred, sponsors had submitted protocols that described highly complex study designs [usually involving legally incapacitated subjects, gene modifications or advanced therapy medicinal products (ATMPs)] but had provided what was considered to be insufficient data to justify their proposed clinical investigations. There were no major changes in the number of submissions between 2013 and 2017, consisting of about 130 Phase I trials per year; rejections making around 2 – 5 trials. About 25% of rejections in these ‘complicated’ studies were as a consequence of the sponsor failing to establish totality of evidence or insufficient clinical pharmacological or mechanistic explanation.

### Agence Nationale de Sécurité du Médicament et des Produits de Santé, France

*Marc Martin*, Deputy Director at the Agence Nationale de Sécurité du Médicament et des Produits de Santé (ANSM, France), opened his presentation by noting that he could not discuss technical issues from the point of view of an assessor. His unit was created to provide the expertise needed to assess applications to conduct early phase clinical trials; this does not include trials with cell or genetic therapies, organs, tissues or cells, vaccines or medical devices. The agency has assessed around 90 submissions and 300 protocol amendments in the first 6 months of 2018. Statistics indicate that the agency provides decisions on submissions within 71 – 80 days in 2017, with intermediate letter sent to sponsors within 70 days (on average). Since May 2018, these delays have been reduced to around 32 days for delivery of intermediate letters and 41 days for decisions, following process optimization. The objective remains to reduce the time further.

The agency is committed to improving the authorization process and facilitating access to innovation for patients. Two fast-track evaluation processes for the early phase trials in patients and for the trials involving a drug that has already been evaluated by the agency have also been initiated by ANSM. Issues that frequently arise from the assessment of the CTA include poorly defined stopping rules, failure to include a sequential approach to inclusion of patient subjects and insufficient information on the pharmacokinetic profiles of IMPs. Submissions also frequently fail to provide sufficient opportunity for data review prior to inter-cohort switch during the trial and where decisions are made they often rely simply on pharmacokinetic data to make decisions on dose escalation.

The agency often requests that sponsors incorporate a Data and Safety Monitoring Board (DSMB) into their study plans. Currently, the EMAs 2006 guideline on the inclusion of data monitoring committees is subject to interpretation and sponsors often request clarification of the agency’s requirements for inclusion of a DSMB (European Medicines Agency Science Medicines Health, 2019). Pharmacovigilance issues also arise frequently, such as there being no formal plan for communication between participants, sponsors and investigators for serious adverse events, Suspected Unexpected Serious Adverse Reactions (SUSARs) or other emerging safety concerns. The consequence seems to be a delay in the period between such episodes and formal notification.

### Státní ústav pro kontrolu léčiv, Czechia

*Ondřej Palán*, from SUKL, the Czech Republic’s State Institute for Drug Control, reviewed historical data on requests submitted to the agency to conduct clinical trials from 2017. Of 628 requests, most studies were not conducted in the Czech republic alone and included requests for scientific advice. Of this total, 5% were for Phase I and 27% were Phase II studies. Critical inadequacies frequently noted during approval review included:

1. Insufficient justification for the doses selected for study;
2. Unclear or undefined age limits;
3. Inappropriately short follow up times; and
4. Having an inadequate number of visits and related quality of examinations.

Concerns were also expressed over the ready ‘availability’ of emergency units that are occasionally not close enough to the study setting.

The revised guideline also emphasizes the importance of non-clinical data from at least two relevant animal species. For modern bioactive compounds, however, it can be difficult to gather relevant data and there may be no acceptable models that can provide appropriate data. Integrated protocols remain a big challenge for regulators who need to ensure that decision points are managed with adequate levels of care.

The Czech agency continues to adopt a risk-based approach, addressing each new submission on a case-by-case approach, especially in the circumstance of adaptive design studies.

### Federal Agency for Medicines and Health Products, Belgium

*Sonja Beken* from the Federal Agency for Medicines and Health Products (FAMHP, Belgium) introduced the attendees to the process of trial approval in Belgium, where in principle, the agency reviews non-clinical/preclinical data (i.e., Investigators Brochure) and quality data of the IMP included in the submission. Separately from the review by the FAMHP, an ethics committee reviews the clinical data and ethical aspects including informed consent forms of each new application. However, this pathway will change slightly with the implementation of the revised FIH guideline, when everything will come under the management of the agency. The main change is the fact that a central body will decide which independent ethics committee (IEC) will review the submission in order to provide further guarantees of the independence of the IEC. The new process is currently being tested. Up till now, about 30 pilot trials of Phase I to IV have been performed according to the new process, sharing the responsibility for clinical trial authorization between the competent authority and the IEC.

The aim of FAMHP is to maintain short turnaround times for study approval, particularly for Phase I trials, providing responses in approximately 15 days. In 2017, there were over 500 new applications for clinical trials and nearly 1,500 trials were run in Belgium in 2016.

Review of the previous clinical trial submission data for Belgium indicates that the reasons for rejections were similar to those reported here by other agencies. These include failure to provide appropriate justification or relevant information to support the highest doses or not conducting non-clinical studies in relevant animal species. Interestingly, some rejections occurred after sponsors had asked the agency for advice but then failed to incorporate the information and direction provided.

For the Belgium authority, *Dr. Beken* reported that the FIH guideline would not change the approach to working of the FAMHP agency and the way it looks into non-clinical and clinical data. The agency is guided by a scientific risk-based approach. Most GNAs issued by FAMHP are administrative in nature, with only a handful being due to scientific issues such as:

(i) Missing data;
(ii) Ill-defined proof of concept;
(iii) Lacking of relevance of the animal species;
(iv) Insufficient non-clinical pharmacology and toxicology data to predict clinical data with adequate confidence;
(v) Absence of a defined maximum dose with justification;
(vi) Inadequate amount of safety monitoring;
(vii) Inappropriate dosing schedule as a function of the IMPs PK profile; and/or
(viii) Use of concomitant medication.

When performing integrated protocols, sometimes conditional approval is possible, i.e., the single ascending dose (SAD) part of a trial may start but the authority require more data before the start of the MAD is permitted. A clinical trial application may also be rejected in cases where prior scientific advice given by EMA or at a national level has not been followed by the sponsor with no adequate reasoning being provided.

### Discussion on Conditional Approval

The audience discussed aspects of conditional approval. A German delegate expressed the opinion, that in case of a conditional approval this would be more likely for integrated protocols in Germany than in other countries. According to *Joop van Gerven* conditional approval is impossible from the legal point of view, however, the authority may ask for additional data while the study proceeds. As there are differences between European countries, *Stefano Ponzano* suggested that specific examples could be discussed at the CTFG Meetings to address different approaches across the European agencies. A training curriculum that focuses on FIH trials is currently being developed by the EMA.

## Delegate Discussion, Questions, and Opinions

### Sentinel Dosing/Staggering of Subjects Within a Multiple-Ascending Dose (MAD) Trial

The sentinel dosing approach was discussed in detail by the audience. Uncertainties exist in the interpretation of the timing of sentinel groups prevail regarding MAD trials. The following questions were raised:

1. Is a sentinel group required for each dose step in a MAD or only for the first group?
2. How long after dosing of the sentinel subjects should the subsequent subjects start?
3. Is this depending on operational feasibility or on PK characteristics of the IMP?

Attendees reported divergent approaches. An overview on the use of sentinel subjects in a MAD setting is summarized in Table 3 and based on answers given by the online tool sli.do.

**Table 3:** An overview on the use of sentinel subjects in a MAD setting.

Question	Respondents	Yes %	No %	It depends
Who did ever implement a sentinel dose group in a MAD part of a trial?	*N* = 63	57	43	n.a.
Do you need a sentinel approach in MAD trials if these doses have already been studied in a SAD trial?	*N* = 68	19	16	65%
Do you need a sentinel approach in MAD trials if they contain a dose escalation beyond the prior SAD trial?	*N* = 46	74	2	24%


The clinical practice of attendees varies between inclusion of sentinel subjects in each cohort (SAD, MAD) or not at all in MAD trials. In general, the audience felt that sentinel dosing in MAD studies is not needed if SAD dose levels covered the MAD levels, but is needed if dose escalation beyond SAD dose levels is intended.

Attendees considered the inclusion of sentinel subjects in each new cohort in the MAD part to be challenging. They preferred a ‘risk-based’ over a ‘checklist’ approach. Further clarification is required on how to define sentinel dosing:

(i) One subject on active medication and one subject on placebo?
(ii) Split a cohort into several groups to reduce the risk (i.e., staggered dosing)?

As a general rule, not all subjects within a cohort should be dosed on the same day. Important safety issues in a MAD trial may occur when steady state has been reached and in case of non-linear pharmacokinetics.

Regulators expressed support for an approach that uses two front-runners (one subject on active medication and one subject on placebo). Depending on the pharmacokinetic half-life of the IMP, the sentinel subjects should be dosed at least 24 – 48 h before the rest of the cohort. Regulators emphasized that according to the revised guideline, omission of sentinel dosing requires justification: ‘Why do people feel there is no risk?’

There was also the question whether the IEC has to give an approval for each dose escalation step in a SAD or a MAD trial if the steps are within the range defined in the trial protocol? *Kirsty Wydenbach* explained how, where the authorities have approved the protocol, no interim report or amendments are required. Attendees asked how far the dose escalation may proceed in an adaptive design trial and whether this would only depend on safety data? *Kirsty Wydenbach* commented that it depends on the dose escalation steps and details specified in the protocol. An attendee reported that Belgian IECs for some FIH trials requested safety reports on individual dose step to remain informed about the decisions on further dose escalation, without necessarily requiring approval (i.e., notification).

The audience felt that this point highlighted differences between European agencies and that the decision procedure requires further harmonization across Europe. Trust should be built between authorities and sponsors as well as investigators. It was proposed that a sponsor personal liability concept (equaling the personal liability defined in the Qualified Person concept) might help to increase trust between stakeholders.

### Maximum Exposure/Supra-Therapeutic Doses

The next point of discussion addressed why - in contrast to what was the common procedure about a decade ago – current practices preclude the determination of the MTD in a FIH trial in healthy subjects. Nevertheless, a supra-therapeutic dose needs to be defined. Safety margins are required regarding therapeutic doses in the transition from healthy subjects to patients. A supra-therapeutic dose is also required for certain types of safety trials (e.g., thorough QT trial). Accordingly, predefining the adequate exposure cap for a FIH trial needs to be carefully considered.

Regulators commented that the MTD is by definition a dose below a toxic dose and thus can only be determined by the observation of a toxic dose level, which is no longer considered acceptable in healthy subjects. A supra-therapeutic dose can be administered to healthy volunteers if an appropriate rationale is provided and the selected dose is clearly below toxic levels. It is imperative to avoid the MTD in healthy subjects.

Yet, often there seems to be a misunderstanding regarding the notion of ‘supra-therapeutic’ dose, this does not need to be, for example, a 10-fold higher dose than the intended therapeutic dose. Recent experience shows a trend for lower maximum doses in FIH trials in healthy subjects. Pharmacokinetic/pharmacodynamic guidance, including the influence of the disease or other patient characteristics and applying a risk-based approach may help to identify the adequate exposure cap.

It is imperative to define maximum exposure in the protocol. Regulators stated they would expect an early phase trial to establish a well-defined top dose in order to ascertain the therapeutic window.

### Dose Escalation Above the NOAEL

Another important question discussed by regulators and audience was whether – in an ascending dose design – an exposure above NOAEL would be acceptable.

Some delegates felt this cannot be answered in general terms but rather requires a risk-based approach, involving a discussion between sponsor and investigator on the IMP and the nature and severity of non-clinical findings as well as the mechanism and clinical ‘monitorability’ of safety/tolerability. The trial protocol should clearly define whether mean exposure of an entire dose group or exposure of an individual subject must not be in excess of NOAEL, precluding further dose escalation. *Stefano Ponzano* pointed out that the FIH guideline in this context indicates individual exposure and *Joop van Gerven* stated that NOAEL is defined on the basis of individual animals. This view was supported by a delegate from the audience, emphasizing that individual exposure should be checked as the number of subjects in a FIH trial is limited. Another delegate disagreed, mentioning that individual animals are taken into account to evaluate toxicology findings, but pharmacokinetic information is typically associated with mean data. To allow a comprehensive understanding of NOAEL data, the individual animal toxicology findings should be related to the individual pharmacokinetic data. The audience felt that – based on all available information – the Principal Investigator needs to be convinced that progression to a higher dose step is safe.

### Relevance of Pharmacodynamic Assays in Non-clinical and Clinical Development

The discussion continued regarding the relevance of pharmacodynamic assays to be used for dose escalation decisions and definition of stopping criteria. What if the appropriate biomarkers are not known or not yet available in FIH trials? In response, *Joop van Gerven* stated that a good pharmacological rationale is the foundation/pillar for the development of a new compound. Therefore, appropriate biomarker/pharmacodynamic measures for the activity of the drug need to be developed and used in non-clinical and clinical studies. Delegates agreed that based on their own experience, the development of biomarkers is time-consuming, expensive, and not always successful. This is particularly the case for disease biomarkers, but it may also be difficult to find reliable pharmacodynamics markers. *Sonja Beken* insisted, if dosing is not based on pharmacological considerations, the results of drug development will not be stringent. *Joop van Gerven* emphasized the importance to characterize the pharmacological activity of a drug. Especially in early phase trials, pharmacodynamic data are very informative and helpful for decision-making, and they reduce the chance of drug development failures. *Stefano Ponzano* agreed every effort must be made to demonstrate the pharmacological effects, even if no appropriate biomarkers are available. *Kirsty Wydenbach* stated the analysis of the pharmacodynamic/pharmacokinetic interaction could also contribute to safety assessments. Some delegates, however, saw limitations to PD-marker guided decisions during conduct of the study as these markers often are only analyzed after the trial is completed. *Joop van Gerven* responded that such limitations often require a more cautious approach, especially for new targets or high-risk compounds.

The audience questioned the regulators whether, in cases where non-clinical studies failed to establish the expected pharmacological characteristics of a new compound, it would be justified to increase the dose in a FIH setting and look at safety and pharmacokinetics only? *Joop van Gerven* suggested that if a marker could not be found, dose escalation may be guided by solely by safety and pharmacokinetics, provided that particular emphasis is placed on predictions of target concentrations based on human predictions of the pharmacologically active concentration range, using good preclinical pharmacodynamic/pharmacokinetic-models (preferably incorporating efficacy and safety) and, if possible, include drug measurements close to the target site (e.g., in the cerebrospinal fluid for drugs acting in the central nervous system).

## Harmonization Between Countries and Strategies of the Industry

At the completion of the forum, attendees were asked about their current experience with competent authorities and about their strategies in facing any perceived regulatory challenge. The online tool *sli.do* was used, answers were provided anonymously.

The data on harmonization between countries suggest the following conclusions:

Applicants experience remarkable differences between European countries. A large proportion of applicants go ‘shopping’ for the agency with the perceived lowest hurdles and the most frequent reason to do this is to save time (Box 1). In response, regulators emphasized that current training initiatives are organized by EMA. Regular CTFG meetings are held to ensure that major aspects of evaluation would be the same, even if some standards differ across Europe. Regulators also expect that with application of the new EU clinical trial regulation harmonization across Europe will increase.

**BOX 1. Applicant differences between European countries. Question:**

According to your experience, do regulators apply the new guidance with the same approach across Europe?

Sixty answers resulted in 12% Yes and 88% No

Question: Do applicants go shopping for the country/agency with the perceived lowest hurdles?

Sixty six answers resulted in 89% Yes and 11% No

Question: What do applicants shop for?

Seventy-one answers were received, multiple answers were allowed.

Pre-clinical benefits: 27%

Clinical benefits: 59%

Ethical benefits: 17%

Quality of advice: 61%

Time-saving 94%

## Conclusion

In closing the meeting it was generally agreed that the necessary changes encompassed by new guidelines included both constructive and disruptive aspects. Sixty-nine delegates took part in the final vote on whether the new FIH guideline is disruptive or constructive: 51% stated it was both constructive and disruptive, 48% decided on constructive, none on disruptive and 1% were still undecided. It was generally accepted that stakeholders need to continue in a process of stakeholder engagement and discussion, particularly on critical safety issues. Such an approach allows partners to adopt a proactive approach to sharing best practice. For example, attendees agreed that a ‘Question and Answer’ document harmonized between the European agencies is required for the sentinel approach and for the selection of supratherapeutic doses.

## Author Contributions

All authors listed have made a substantial, direct and intellectual contribution to the work, and approved it for publication.

## Conflict of Interest Statement

TH is employed by the company, Niche Science & Technology Ltd. The remaining authors declare that the research was conducted in the absence of any commercial or financial relationships that could be construed as a potential conflict of interest.

## Footnotes

1 www.eufemed.eu

2 www.sli.do
